# Identifying an immunogenic cell death-related gene signature contributes to predicting prognosis, immunotherapy efficacy, and tumor microenvironment of lung adenocarcinoma

**DOI:** 10.18632/aging.205705

**Published:** 2024-04-03

**Authors:** Xue Li, Dengfeng Zhang, Pengfei Guo, Shaowei Ma, Shaolin Gao, Shujun Li, Yadong Yuan

**Affiliations:** 1 Department of Respiratory and Critical Care Medicine, The Second Hospital of Hebei Medical University, Shijiazhuang 050000, China; 2 Department of Thoracic Surgery, The Second Hospital of Hebei Medical University, Shijiazhuang 050000, China; 3 Department of Gastrointestinal Surgery, The Second Hospital of Hebei Medical University, Shijiazhuang 050000, China

**Keywords:** immunogenic cell death, lung adenocarcinoma, prognosis, tumor microenvironment, immunotherapy

## Abstract

Background: Immunogenic cell death (ICD) is a regulated form of cell death that triggers an adaptive immune response. The objective of this study was to investigate the correlation between ICD-related genes (ICDGs) and the prognosis and the immune microenvironment of patients with lung adenocarcinoma (LUAD).

Methods: ICD-associated molecular subtypes were identified through consensus clustering. Subsequently, a prognostic risk model comprising 5 ICDGs was constructed using Lasso-Cox regression in the TCGA training cohort and further tested in the GEO cohort. Enriched pathways among the subtypes were analyzed using GO, KEGG, and GSVA. Furthermore, the immune microenvironment was assessed using ESTIMATE, CIBERSORT, and ssGSEA analyses.

Results: Consensus clustering divided LUAD patients into three ICDG subtypes with significant differences in prognosis and the immune microenvironment. A prognostic risk model was constructed based on 5 ICDGs and it was used to classify the patients into two risk groups; the high-risk group had poorer prognosis and an immunosuppressive microenvironment characterized by low immune score, low immune status, high abundance of immunosuppressive cells, and high expression of tumor purity. Cox regression, ROC curve analysis, and a nomogram indicated that the risk model was an independent prognostic factor. The five hub genes were verified by TCGA database, cell sublocalization immunofluorescence analysis, IHC images and qRT-PCR, which were consistent with bioinformatics analysis.

Conclusions: The molecular subtypes and a risk model based on ICDGs proposed in our study are both promising prognostic classifications in LUAD, which may provide novel insights for developing accurate targeted cancer therapies.

## INTRODUCTION

In 2020, lung cancer contributed to 2.2 million new cases and 1.8 million deaths globally, positioning it as the second most frequently diagnosed cancer (11.4%) and the leading cause of cancer-related mortality (18.0%) [1]. Non-small cell lung cancer (NSCLC) represents the majority, constituting 85% of all lung cancer diagnoses, with lung adenocarcinoma (LUAD) emerging as a prominent histological subtype within NSCLC [2]. Early-stage LUAD often presents with nonspecific clinical manifestations, resulting in challenges in early detection. As the disease progresses, LUAD tends to manifest local infiltration and distant metastases, leading to a dismal prognosis with an overall 5-year survival rate of less than 20% [3]. Targeted therapy and immunotherapy are among the main approaches for LUAD treatment, and although both have achieved good efficacy [4], the clinical benefit to patients remains limited [5]. The common clinical indicators used to predict the prognosis of LUAD include tumor size, metastasis, and tumor mutational burden (TMB); however, tumors are highly heterogeneous, and the treatment effects and prognosis still vary greatly, even in patients with the same TNM stage [6]. Relying on these indicators alone is not always sufficient to accurately predict patient prognosis. Therefore, it is crucial to clarify the molecular mechanisms and characteristics of LUAD and to identify biomarkers that can predict the prognosis of patients with LUAD.

Immunogenic cell death (ICD) represents a distinct mechanism of cellular demise whereby a “risk signal” is unveiled through the release of tumor-associated antigens (TAAs) and tumor-specific antigens (TSAs), thereby inciting an immune response within the organism [7, 8]. Central to the phenomenon of ICD is the emergence and/or escalation of damage-associated molecular patterns (DAMPs), comprising entities such as adenosine triphosphate (ATP), calreticulin (CRT), and high mobility group box 1 (HMGB1) [9]. ICD occurs when multiple chemotherapeutic drugs with specific cytotoxicity (such as oxaliplatin and taxol) or physical therapy (radiation and photodynamic therapy) are used to treat tumors, and tumor cells change from non-immunogenic to immunogenic, in which they recruit and activate immune cells to stimulate or enhance the anti-tumor immune response [10–13]. As a critical component of antitumor immunotherapy, further research into and elucidation of the mechanisms involved in the occurrence of ICD are particularly important for tumor treatment, as well as the development of tumor vaccines.

In this study, 1315 LUAD samples were divided into three ICD-related subtypes based on 31 ICD-related genes (ICDGs), and the survival and immune microenvironment differences among the subtypes were explored. Additionally, an ICD prognostic signature was established to predict the immune microenvironment, prognosis, and response to immunotherapy in patients with LUAD. The results indicate that the prognostic signature can be used to evaluate prognosis and reflect the efficacy of immunotherapy in LUAD.

## MATERIALS AND METHODS

### Dataset and preprocessing

Samples with survival status unavailable and an overall survival (OS) of fewer than 30 days were omitted from analysis. The RNA-Seq data pertaining to patients with LUAD were retrieved from the TCGA database, formatted in TPM, and log-transformed. This dataset comprised 490 tumor samples and 59 normal samples. Additionally, the GSE72904 and GSE68465 datasets were acquired from the GEO database, accompanied by their respective platform files for annotation, and were designated as validation cohorts. Subsequently, survival analysis encompassed 490 patients from TCGA-LUAD, 386 from GSE72904, and 210 from GSE68465. To mitigate batch effects, the combat function within the “sva” package was employed to preprocess GSE72904, GSE68465, and TCGA-LUAD datasets, culminating in the formation of a unified cohort referred to as the meta-cohort. Notably, IMvigor, PRJEB23709, GSE78220, GSE91061, GSE100797, GSE165252, GSE35640, GSE135222, GSE126044, Nathanson, and the VanAllen datasets constitute immunotherapy cohorts, encompassing clinical information inclusive of responses to immunotherapy. Immunofluorescence staining of A-431 cells and immunohistochemistry (IHC) images were obtained from the HPA database. Both somatic mutation and copy number variation (CNV) data were obtained from the TCGA-LUAD cohort. In addition, 31 ICDGs that were annotated in the TCGA cohort were identified from the relevant literature [14]: IFNA1, IFNG, CXCR3, NT5E, PRF1, IL1R1, BAX, P2RX7, ENTPD1, IL17RA, EIF2AK3, TNF, CASP8, HSP90AA1, TLR4, IL10, HMGB1, NLRP3, FOXP3, CALR, IFNB1, MYD88, IL17A, ATG5, LY96, PDIA3, IFNGR1, PIK3CA, CASP1, IL6, and IL1B. In brief, our literature search involved multiple databases, including PubMed, and Web of Science, to ensure comprehensive coverage. We used a combination of keywords and phrases related to “Immunogenic Cell Death”, “ICD”, and “ICD-Associated Genes”. We applied filters to select studies published in the last ten years, focusing on those that clearly identified genes associated with ICD in the context of cancer.

Moreover, GSE127465 is a single-cell sequencing set of LUAD, and its processing pipeline is from the TISCH database. We downloaded the expression profiles and cell annotation files processed by the TISCH database, and based on the ICDGs participating in the risk signature, we scored each malignant cell using “AddModuleScore” function and used the median value to obtain malignant cells with high risk scores, and then finally implemented the default parameters in the “cell chat” package to perform cellular communication between malignant cells and other cells.

### Consensus clustering

Unsupervised consensus clustering analysis was conducted based on prognostic ICDGs (*p* < 0.05). Principal component analysis (PCA) was then utilized to assess the relative independence of each subtype from the others. The determination of the optimal number of clusters was carried out employing the R package “ConsensusClusterPlus”, with 1,000 replicates and a pltem parameter set to 0.8 to assess subtype stability. OS disparities among clusters were evaluated via the Kaplan-Meier (KM) method, with the log-rank test employed to discern survival differences. Distributional disparities of categorical data across clusters were assessed using the chi-squared test. Furthermore, differentially expressed genes (DEGs) among distinct molecular subtypes were investigated utilizing the “limma” package.

### Enrichment analysis

The “clusterProfiler” package within the R software environment facilitated GO annotation and KEGG pathway analysis. Significance in pathway enrichment was determined based on a threshold of *P*-value < 0.05 and *q*-value < 0.05. Distinctions in biological pathways among subtypes were evaluated through Gene Set Enrichment Analysis (GSEA). Specifically, the c2.cp.kegg.v7.4.symbols.gmt served as the reference gene set, with a FDR threshold set at < 0.05 for screening purposes.

### Immune analysis

Immune cell analysis incorporated a comprehensive array of algorithms, including TIMER, CIBERSORT, QUANTISEQ, MCP-counter, XCELL, and EPIC, to assess the diverse immune cell infiltration across distinct samples. Furthermore, the ESTIMATE algorithm was employed to compute stromal, ESTIMATE, and immune scores, thereby offering insights into the tumor microenvironment status. Following the classification proposed by Thorsson et al. [15], which categorized solid tumors in TCGA into six distinct immune expression signature subtypes, namely wound healing (Immune C1), IFN-gamma dominant (Immune C2), inflammatory (Immune C3), lymphocyte depleted (Immune C4), immunological quiet (Immune C5), and TGF-beta dominant (Immune C6), our analysis provided a nuanced understanding of immune dynamics within the tumor milieu.

### Construction and validation of the ICD prognostic signature

The TCGA-LUAD cohort was utilized for modeling purposes, while the GSE68465 cohort was employed for external validation. Redundant genes within the ICDGs were eliminated using the least absolute shrinkage and selection operator (LASSO) regression method. Subsequently, correlation coefficients and gene expression values were obtained through multivariate Cox regression analysis to establish a risk score formula. Subsequent categorization of patients into high- and low-risk subgroups was based on the median value derived from the risk score formula. Univariate and multivariate Cox regression analyses were employed to evaluate the prognostic significance of the risk score within both the primary dataset and an external validation cohort. Receiver operating characteristic (ROC) curves were generated, and the area under the curve (AUC) was computed utilizing the “survivalROC” package to ascertain the predictive performance of the clinical signature. Furthermore, a prognostic nomogram was formulated utilizing the independent prognostic factors discerned through multivariate Cox regression. Subsequently, the nomogram’s performance was verified through calibration analysis.

### Mutation and drug sensitivity analysis

The semi-inhibitory concentration (IC50) values were determined utilizing the “pRRophetic” package within the R software environment, with chemotherapy agents sourced from the Genome of Drug Sensitivity in Cancer (GDSC) database. Mutational disparities were illustrated using the “oncoplot” function of the “Maftools” R package.

### Protein expression validation

Immunohistochemistry (IHC) Validation: We used immunohistochemical staining images from the Human Protein Atlas (HPA) database, available at v19.3.proteinatlas.org [16], to confirm the expression of the critical genes under investigation in both LUAD and adjacent normal tissue specimens. The evaluation of IHC images in the HPA database involved a comprehensive assessment of staining characteristics, intensity, quantity, and location with regard to individual genes (Detailed information about the statistical methods used for analyzing IHC images is available at the following web address: https://www.proteinatlas.org/about/assays+annotation#ih_annotation).

### Quantitative real-time PCR (qRT-PCR)

Ten tumor tissue samples and adjacent normal lung tissue samples were procured from patients diagnosed with LUAD who underwent tumor resection at the Thoracic Surgery Department of The Second Hospital of Hebei Medical University. Ethical approval for sample collection was granted by the Medical Ethics Committee of the hospital. The experimental methodology was adapted from our previous work [17]. Briefly, total RNA was isolated from LUAD tissue samples and their corresponding normal tissues using the TRIzol reagent (Invitrogen, Carlsbad, CA, USA), following the manufacturer’s instructions. The quality and quantity of the extracted RNA were evaluated utilizing the NanoDrop ND-1000 Spectrophotometer. Total RNA (1 μg) underwent cDNA synthesis employing the High-Capacity cDNA Reverse Transcription Kit (Applied Biosystems, Foster City, CA, USA), adhering meticulously to the manufacturer’s protocol. qRT-PCR assays utilized the PowerUp SYBR Green Master Mix (Applied Biosystems) on the QuantStudio 5 Real-Time PCR System (Applied Biosystems). Primer sequences for target ICD-related genes and the endogenous control (GAPDH) were crafted utilizing the Primer Bank database. The 2^−ΔΔCT^ method was employed to compute the relative expression levels of the target genes, with GAPDH serving as the normalization control. Triplicate runs of each sample were executed to ensure result precision. Primer efficiency was validated by generating standard curves through serial dilutions of cDNA, and specificity was confirmed by analyzing melting curves post-amplification.

The primer sequences were as follows:

GAPDH Forward: 5′-GGAGCGAGATCCCTCCAAAAT-3′, Reverse: 5′-GGCTGTTGTCATACTTCTCATGG-3′, NT5E Forward: 5′-GCCTGGGAGCTTACGATTTTG-3′, Reverse: 5′-TAGTGCCCTGGTACTGGTCG-3′, HSP90AA1 Forward: 5′-AGGAGGTTGAGACGTTCGC-3′, Reverse: 5′-AGAGTTCGATCTTGTTTGTTCGG-3′, EIF2AK3 Forward: 5′-ACGATGAGACAGAGTTGCGAC-3′, Reverse: 5′-ATCCAAGGCAGCAATTCTCCC-3′, PIK3CA Forward: 5′-CCACGACCATCATCAGGTGAA-3′, Reverse: 5′-CCTCACGGAGGCATTCTAAAGT-3′, P2RX7 Forward: 5′-TATGAGACGAACAAAGTCACTCG-3′, Reverse: 5′-GCAAAGCAAACGTAGGAAAAGAT-3′.

### Statistical analysis

Statistical analyses were conducted using R software (version 4.0.1). The preceding section delineates comprehensive statistical methodologies employed for transcriptome data processing. Statistical significance was defined as a *p*-value below 0.05.

## RESULTS

### Landscape of ICDGs in LUAD

**Figure 1:**
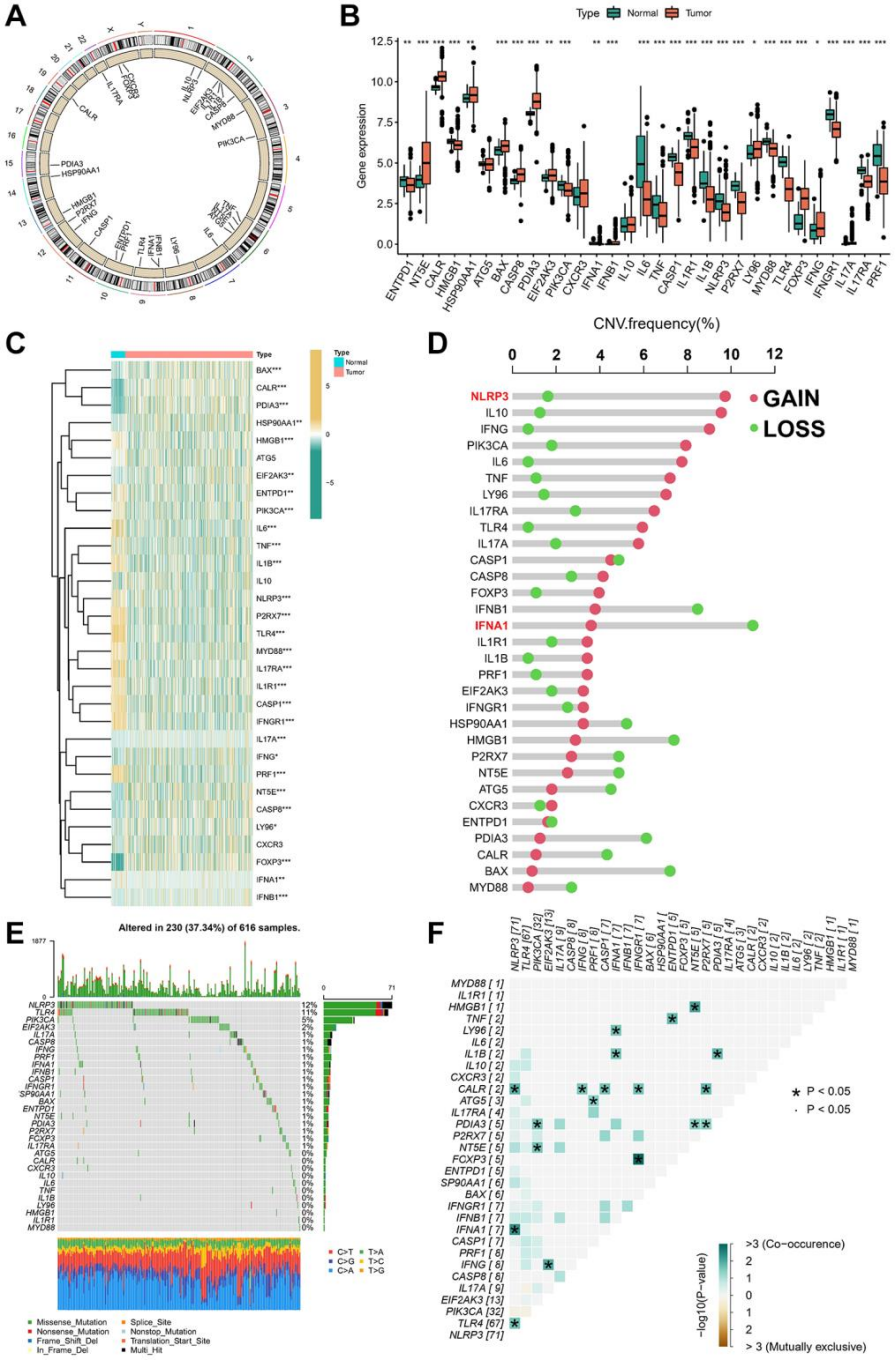
**Landscape of 31 ICDGs in LUAD.** (**A**) Circus plots of chromosome distributions of ICDGs. (**B**) Expression distributions of ICDGs between LUAD and normal tissues. (**C**) Heatmap shows 31 ICDGs expression profiles among normal and LUAD samples. (**D**) The CNV mutation frequency of ICDGs. (**E**) Somatic mutation spectrums of ICDGs. (**F**) Correlation between 31 ICDGs in TCGA-LUAD. ^*^p < 0.05, ^**^p < 0.01, ^***^p < 0.001.

In this study, 31 ICDGs were identified in TCGA cohort, and the distribution of ICDGs on chromosomes was shown in Figure 1A. We investigated the mRNA expression levels of ICDGs between normal and LUAD samples; the ICDGs other than ATG5, IL10, and CXCR3 were significantly differentially expressed between different samples (Figure 1B, 1C). The CNV mutation frequency indicated that CNV alterations were prevalent in ICDGs, with NLRP3 concentrating on copy number amplification and IFNA1 having the highest frequency of CNV deletion (Figure 1D). As is evident from Figure 1E, 230 of 616 (37.34%) LUAD samples show genetic mutations, with NLRP3 showing the highest mutation frequency. Further analysis revealed a significant mutational co-occurrence relationship between NLRP3 and TLR4, IFNA1, and CALR (Figure 1F). This analysis indicates that ICDGs are highly heterogeneous between normal and LUAD samples, suggesting that the expression imbalance of ICDGs plays a crucial role in the occurrence and progression of LUAD.

### Identification of molecular subtypes based on ICDGs

**Figure 2:**
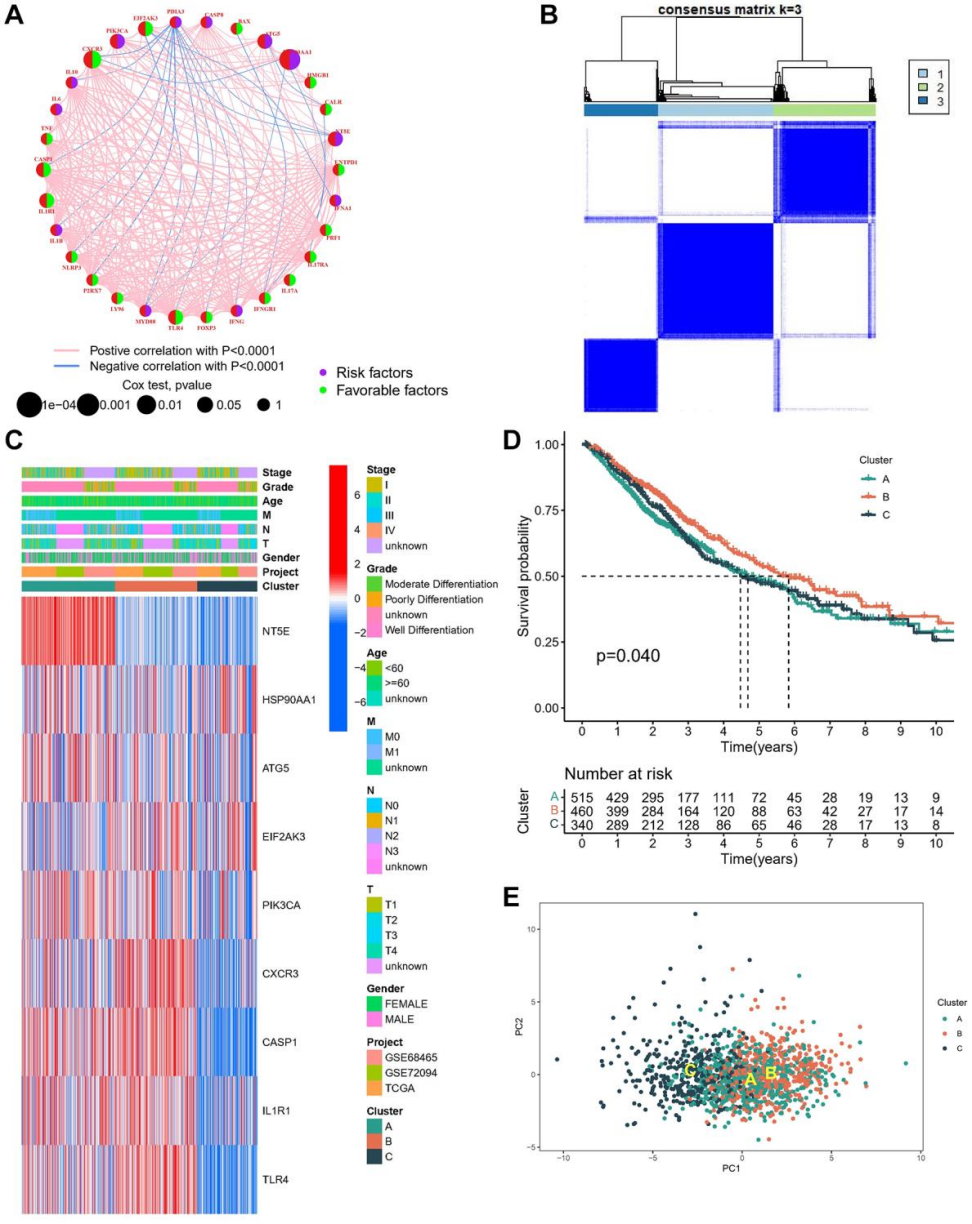
**Identification of molecular subtypes based on ICDGs.** (**A**) The interaction of expression on ICDGs in LUAD. (**B**) Consensus clustering matrix at K = 3. (**C**) Heatmap of the nine genes between the three clusters and the correlations of the clusters and clinical parameters. (**D**) KM curve of the survival difference between the three clusters. (**E**) PCA plot for the three clusters.

The correlation network of ICDGs interactions, regulator relationships, and their survival significance in LUAD patients is presented in Figure 2A. Most ICDGs exhibited significant correlations and had good prognostic ability. The GSE72904, GSE68465, and TCGA-LUAD datasets were combined into a meta-analysis using the combat algorithm, and 1315 LUAD patients were included. The prognostic value of 23 ICDGs for patients with LUAD was revealed by KM survival analysis and log-rank test, distinguished by the best cut-off value of each group (Supplementary Figure 1).

The meta-cohort underwent screening for ICDGs utilizing univariate Cox regression analysis, identifying nine genes as prognostically significant ICDGs. Subsequently, to elucidate the association between the expression profiles of ICDGs and subtypes of LUAD, a consensus clustering analysis was conducted to categorize LUAD patients based on the expression levels of these prognostic ICDGs. Optimal clustering was observed at K = 3 (Figure 2B), leading to the classification of LUAD patients into distinct subtypes labeled as A (*n* = 515), B (*n* = 460), and C (*n* = 430) across the entire cohort. A heatmap depiction illustrates the distribution of clinical attributes across different clusters, revealing significant upregulation of most genes in subtypes A and B (Figure 2C). Subsequent prognostic evaluation demonstrated a noteworthy survival advantage for subtype B within the molecular subtypes (Figure 2D). PCA also confirmed the relative discreteness of the three molecular subtypes (Figure 2E).

### Immune microenvironment and functional enrichment analysis in molecular subtypes

**Figure 3:**
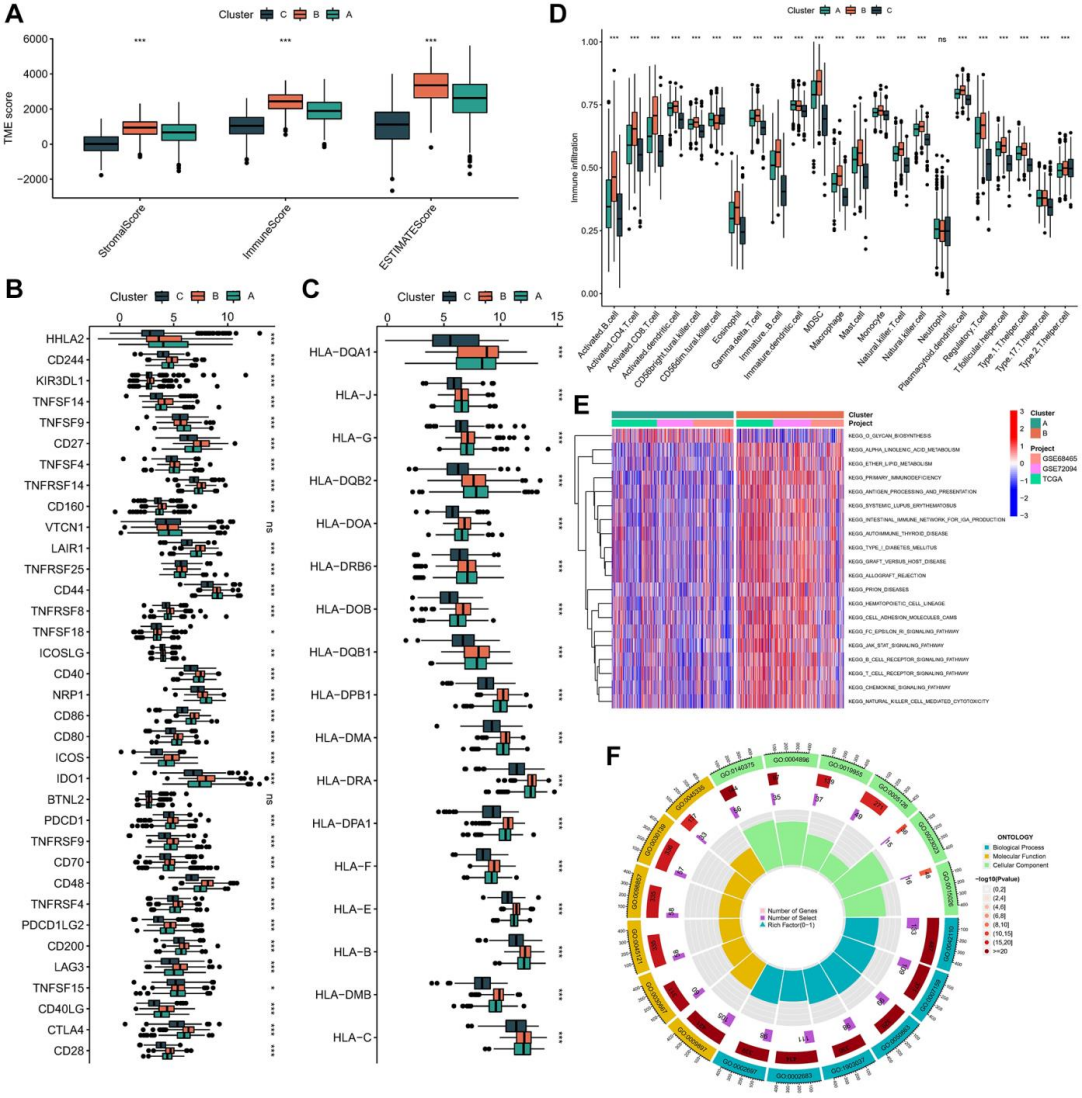
**Immune microenvironment and functional enrichment analysis in molecular subtypes.** (**A**) The difference in stromal score, ESTIMATE score, and immune score between different molecular subtypes. (**B**) Expression of immune checkpoints between different molecular subtypes. (**C**) Expression level of HLA genes between different molecular subtypes. (**D**) The infiltration of immune cells in TME in 3 subtypes. (**E**) The GSVA pathway enrichment analysis between different subtypes. (**F**) GO enrichment analysis results of 1033 DEGs. ns not significant, ^*^p < 0.05, ^**^p < 0.01, ^***^p < 0.001.

The ESTIMATE algorithm unveiled a notably elevated immune score within subtype B (Figure 3A). Furthermore, our investigation elucidated the differential expression of HLA molecules and ICI mRNA across distinct molecular subtypes. Notably, subtype B showcased heightened mRNA expression levels across several ICIs, including CD27, TNFSF4, and TNFRSF14 (Figure 3B). Similarly, subtype B exhibited increased mRNA expression levels across various HLAs, such as HLA-DQA1, HLA-J, HLA-G, and HLA-DQB2 (Figure 3C). Through GSEA, we delineated the TME status among the molecular subtypes, revealing subtype B’s inclination towards a “hot” tumor phenotype, characterized by a more dynamic TME (Figure 3D). Based on these findings, we posited that subtype B likely embodies hot tumor characteristics and stands to derive greater benefits from immunotherapeutic interventions.

In order to elucidate the biological characteristics of these distinct isoforms, we conducted GSVA enrichment analysis. Subtype B exhibited a notably heightened level of enrichment when juxtaposed with subtypes A and C. Our GSVA enrichment analysis unveiled that subtype B demonstrated significant enrichment in pathways associated with immune system activation, encompassing cytokine receptor interaction, chemokine signaling, T and B cell receptor signaling, natural killer cell-mediated cytotoxicity, antigen processing and presentation, as well as epsilon and JAK-STAT signaling pathways (Figure 3E and Supplementary Figure 2A, 2B).

Furthermore, a total of 1033 DEGs were discerned across the three subtypes of LUAD (Supplementary Figure 2C and Supplementary Table 1). Subsequently, these DEGs underwent GO and KEGG enrichment analyses using the “clusterProfiler” tool (Figure 3F, Supplementary Figure 2D). The foremost KEGG signaling pathways encompassed viral protein interactions with cytokines and cytokine receptors, Th17 cell differentiation, hematopoietic cell lineage, Th1 and Th2 cell differentiation, and cytokine-cytokine receptor interactions (Supplementary Tables 2 and 3). These findings offer novel avenues for investigating the potential functions of ICDGs in LUAD.

### Construction and validation of the ICD prognostic signature

**Figure 4:**
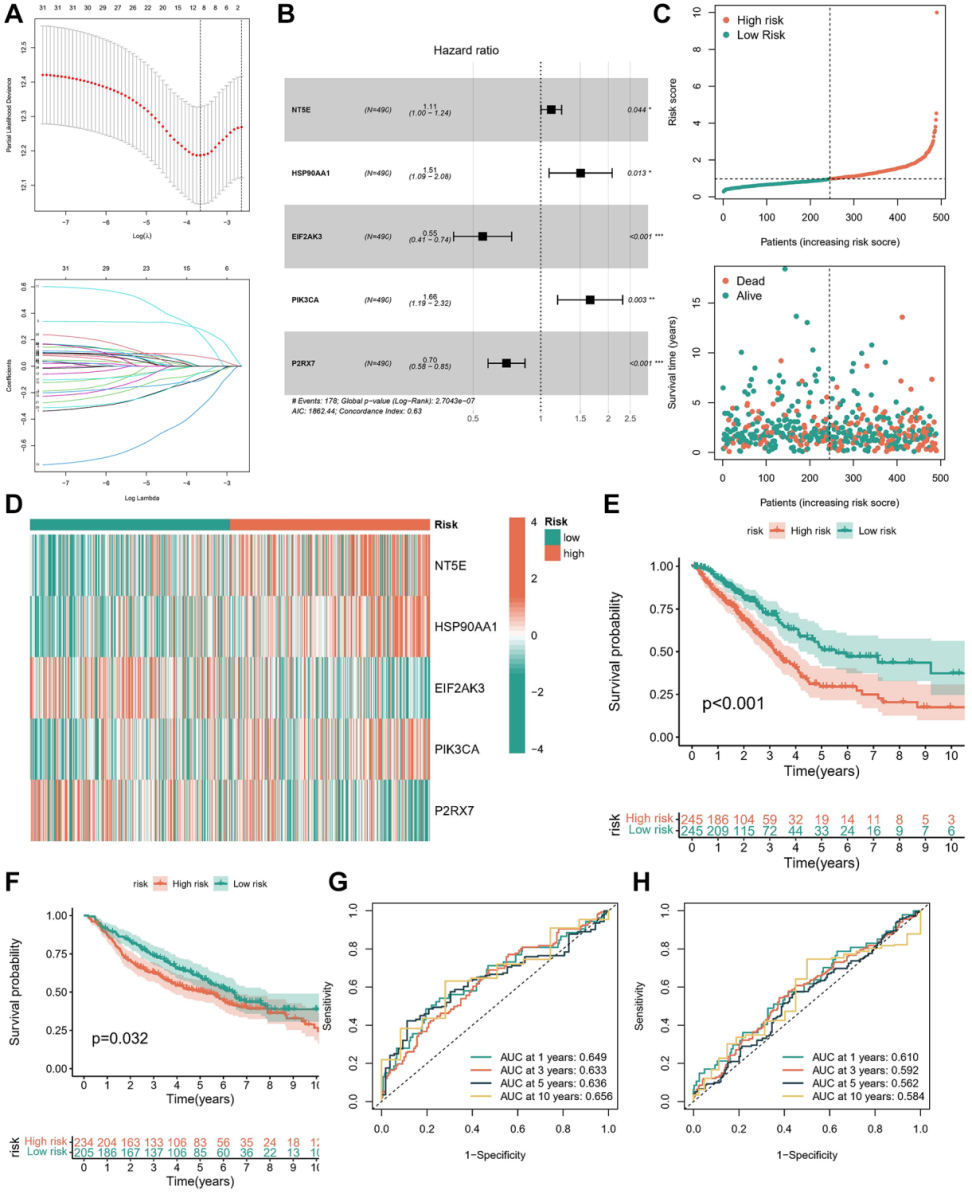
**Construction and validation of the ICD prognostic signature.** (**A**) LASSO Cox regression analysis of ICDGs. (**B**) Forest plot of the five target genes that compose the ICD signature. (**C**) Risk score distribution plot showed the distribution of high-risk and low-risk LUAD patients. Scatter plot showed the correlation between the survival status and risk score of LUAD patients. (**D**) Heatmap for the expression of five crucial genes in low-risk and high-risk subgroups. The survival analysis in TCGA cohort (**E**) and GSE68465 cohort (**F**). ROC curves indicated the predictive efficiency of the prognostic signature in TCGA cohort (**G**) and GSE68465 cohort (**H**). ^*^p < 0.05, ^**^p < 0.01, ^***^p < 0.001.

The training cohort utilized in this study was derived from TCGA, wherein OS served as the primary outcome measure. Initial filtering of redundant genes was conducted through LASSO regression analysis (Figure 4A). Subsequently, multifactorial Cox regression analysis was employed to determine the coefficients associated with each gene (Figure 4B and Supplementary Table 4). Following these analyses, a distinctive signature comprising five genes was identified. The risk score for the ICDG signature was calculated using the following formula: Risk score = (0.1087 × expression level of NT5E) + 0.4088 × expression level of HSP90AA1) + (−0.5949 × expression level of EIF2AK3) + (0.5066 × expression level of PIK3CA) + (−0.3506 × expression level of P2RX7).

In accordance with the median risk score observed within the TCGA cohort, we categorized patients with LUAD into distinct subgroups denoted as low-risk and high-risk. Analysis of the risk status map alongside the survival distribution map revealed a notably diminished prognosis among LUAD patients exhibiting higher risk scores (Figure 4C). Our investigation unveiled significant overexpression of NT5E, HSP90AA1, and PIK3CA within the high-risk subgroup, as evidenced by the ICD signature, while EIF2AK3 and P2RX7 exhibited significant upregulation within the low-risk subgroup (Figure 4D). Furthermore, survival analysis coupled with ROC curve assessment illustrated the robust prognostic capability of the signature within both the TCGA-LUAD (Figure 4E, 4G) and GSE68465 cohorts (Figure 4F, 4H), wherein patients classified within the high-risk subgroup displayed poorer survival outcomes.

### Construction and evaluation of novel nomogram

**Figure 5:**
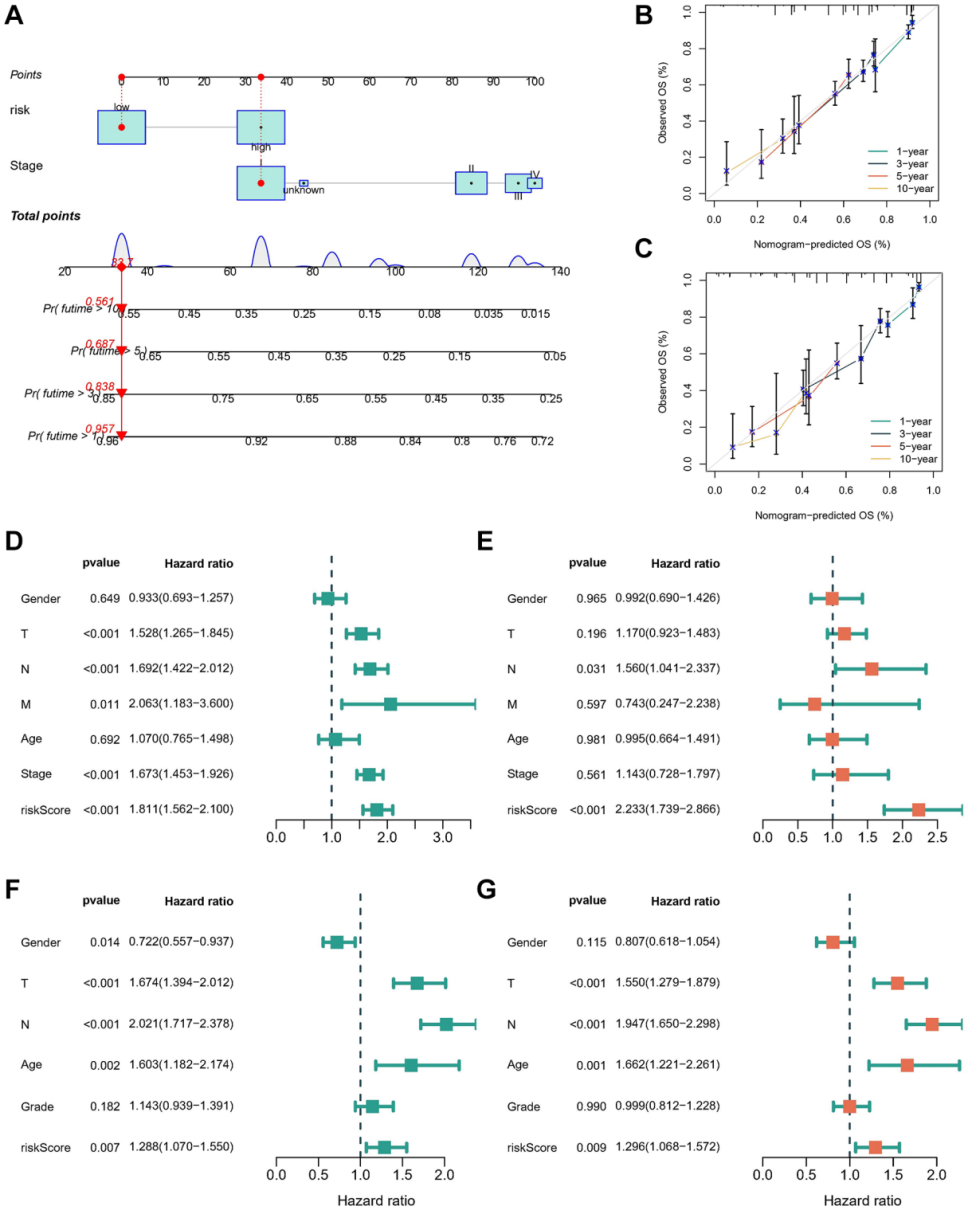
**Construction and evaluation of the novel nomogram.** (**A**) The nomogram for predicting the survival probability of LUAD patients. The calibration plots of the nomogram for predicting OS probability in TCGA cohort (**B**) and GSE68465 cohort (**C**). Univariate (**D**) and multivariate (**E**) Cox analyses for the signature-based risk score and other clinical features in TCGA cohort. Univariate (**F**) and multivariate (**G**) Cox analyses for the signature-based risk score and other clinical features in GSE68465 cohort.

We developed a nomogram integrating the risk score and stage to facilitate the clinical application of our findings (Figure 5A). Calibration curves demonstrated the robust predictive capability of the nomogram based on the risk score in both the TCGA-LUAD (Figure 5B) and GSE68465 cohorts (Figure 5C), closely aligning with the true curve. To assess the risk score’s independence as a prognostic indicator for LUAD, univariate and multivariate Cox regression analyses were conducted in the TCGA-LUAD cohort (Figure 5D, 5E) and GSE68465 cohorts (Figure 5F, 5G). The analyses consistently affirmed the risk score’s status as an independent prognostic predictor (all *p* < 0.01). These findings underscore the efficacy of the prediction model in anticipating OS outcomes, thus warranting its utilization for guiding clinical interventions.

### Immune characteristics in different risk subgroups

**Figure 6:**
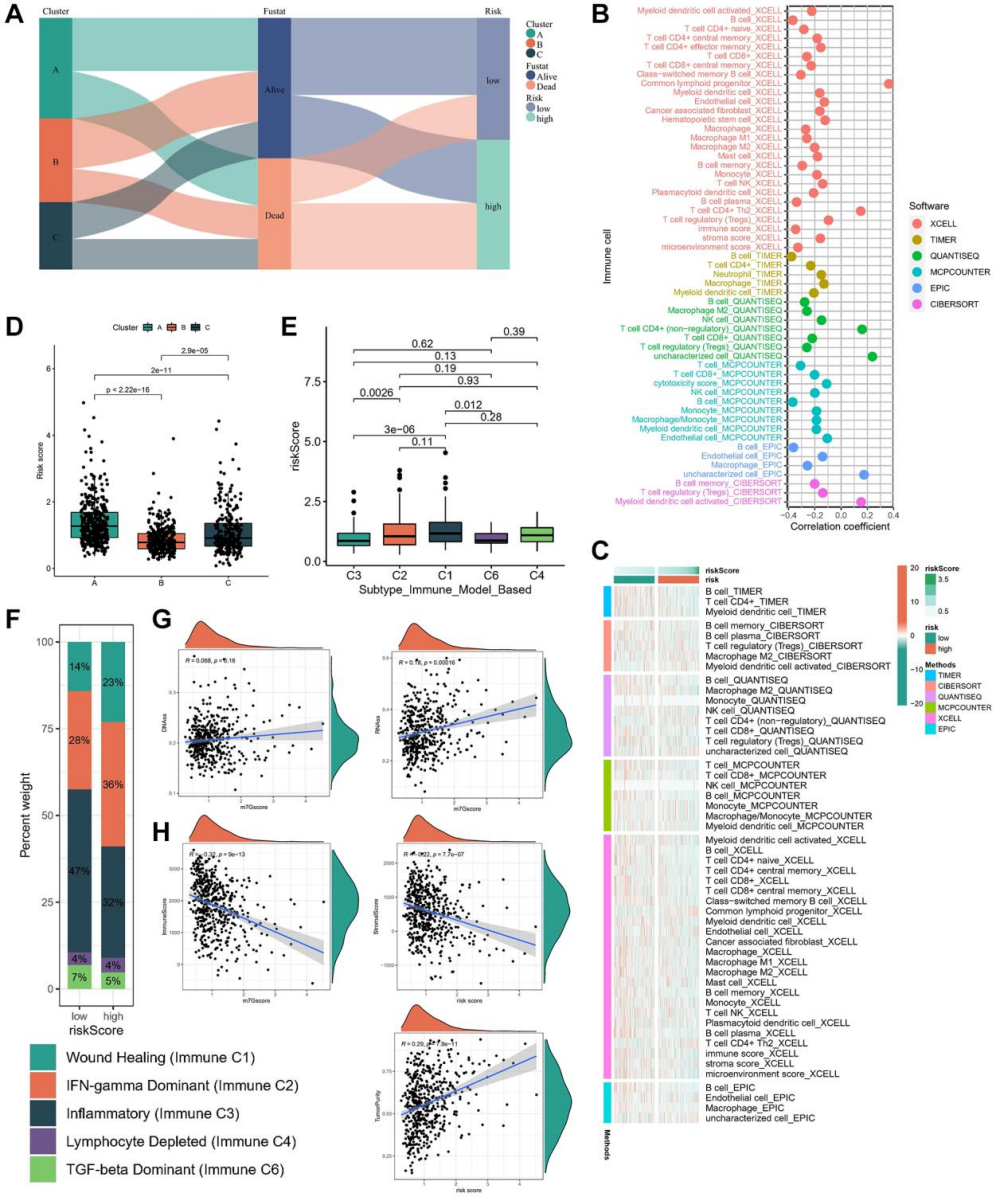
**Immune characteristics in different risk subgroups.** (**A**) The sankey diagram of the relationship among the clusters, risk score and survival state. The correlation of tumor-infiltrating cells with risk score using 6 algorithms. (**B**) Heatmap. (**C**) lollipop plot. (**D**) Risk score levels of cluster A/B/C subtypes. (**E**, **F**) Relationships between risk score and immune subtypes. (**G**) The correlation between risk score and stemness index. (**H**) The correlation between risk score and TME scores.

The Sankey diagram depicted a robust correlation between risk subgroups and molecular subtypes, with a notable survival trend observed among most patients in subtype B and low-risk subgroups (Figure 6A). To elucidate the discriminative capacity of the risk subgroup concerning the TME and its potential application in immunotherapeutic strategies, we employed a comprehensive array of algorithms (TIMER, CIBERSORT, QUANTISEQ, MCP-counter, XCELL, and EPIC) to assess the infiltration levels of immune cells across diverse samples. As anticipated, the abundance of cytotoxic immune cells, including CD4+ T and CD8+ T cells, exhibited a positive correlation with increasing risk scores (Figure 6B), while variations in the distribution of immune cells were observed among distinct risk subgroups (Figure 6C). Furthermore, patients categorized under subtype B demonstrated lower risk scores (Figure 6D). Thorsson et al. [15] delineated six distinct immune expression signature subtypes by analyzing the gene expression profiles across all solid tumors within TCGA. These subtypes are characterized as follows: wound healing (Immune C1), IFN-gamma dominant (Immune C2), inflammatory (Immune C3), lymphocyte depleted (Immune C4), immunological quiet (Immune C5), and TGF-beta dominant (Immune C6). Our analysis revealed notable variations in the expression levels of the risk score across these subtypes, with the lowest expression observed in the C6 subtype and the highest expression observed in the C1/C2 subtype (Figure 6E). Additionally, it was observed that the low-risk subgroup exhibited a relatively higher proportion of the C3 subtype (Figure 6F). Given the pivotal influence of the stemness index on immunotherapeutic outcomes, our correlation analysis revealed a positive association between the risk score and DNAss as well as RNAss (Figure 6G). Subsequently, an investigation into the relationship between the risk score and the TME score was conducted. Our findings indicate an inverse correlation between the risk score and stromal and immune scores, while a positive correlation was observed with tumor purity (Figure 6H). These observations underscore an immune activation profile within the low-risk subgroup, suggesting its potential suitability for immunotherapeutic interventions.

### Association between risk score and response to immunotherapy and chemotherapy

**Figure 7:**
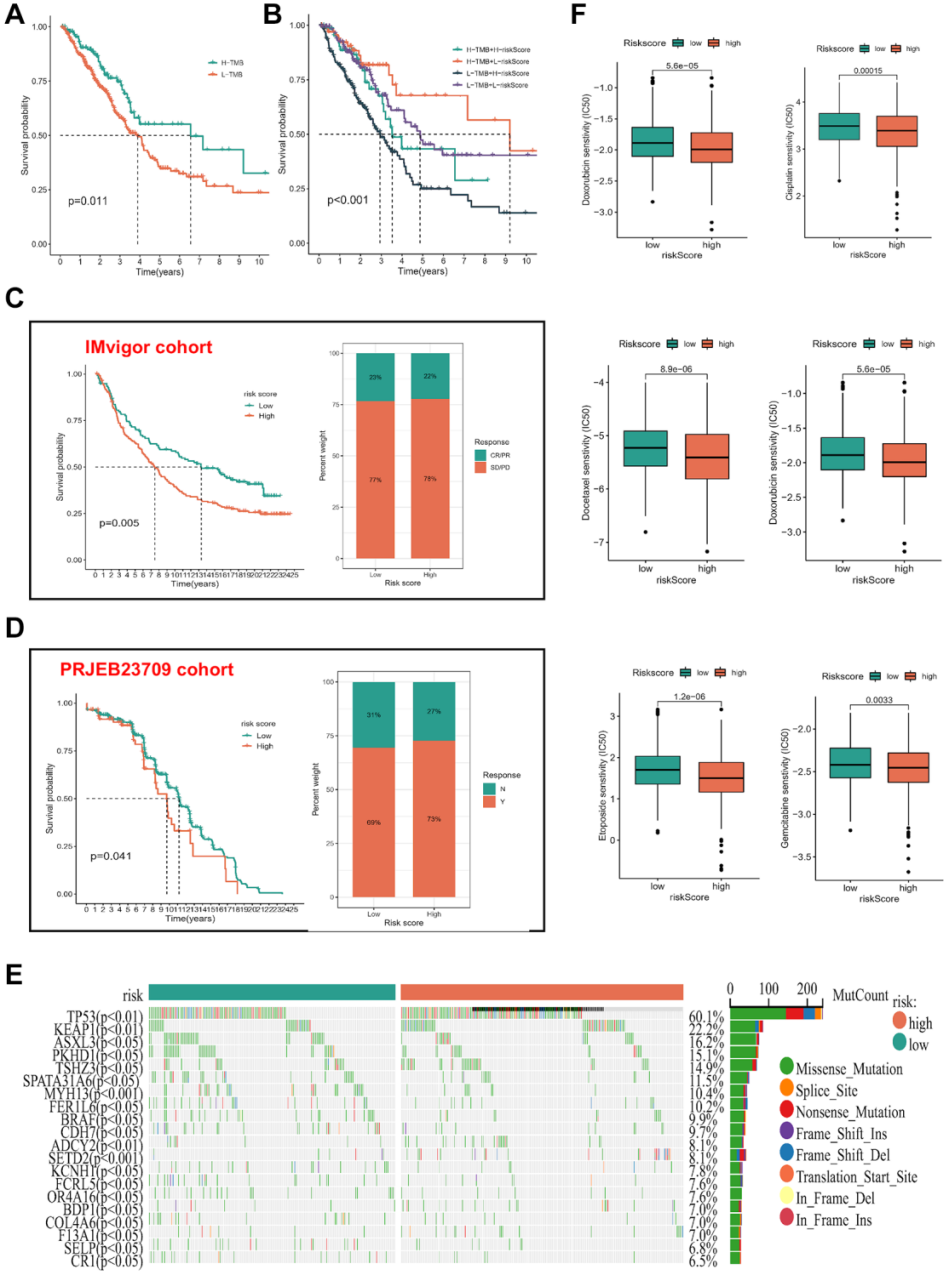
**Association of risk subgroup with therapy in LUAD patients.** (**A**) KM survival analysis of TMB. (**B**) Effects of the risk score combined with TMB on the overall survival. (**C**, **D**) The survival status and immunotherapy reflection of patients in the high- and low-risk subgroups in three immunotherapy cohorts. Abbreviations: CR: complete response; PR: partial response; SD: stable disease; PD: progressive disease. (**E**) Tumor mutation situations in different risk score subgroups. (**F**) The boxplot of sensitivity of common chemotherapy drugs between high- and low-risk subgroups.

Multiple investigations have underscored the significance of TMB as a pivotal prognostic marker for the tumor immune response, correlating high TMB levels with enhanced response rates and superior outcomes in immunotherapy among patients [18–20]. To delve into the genetic alterations of ICDGs in LUAD, a comprehensive TMB analysis was conducted on the risk score. The results delineated an extended survival duration within the high-TMB cohort compared to their low-TMB counterparts (Figure 7A). Furthermore, the amalgamation of high TMB and low risk scores exhibited the most pronounced survival advantage (Figure 7B), accentuating the synergistic effect of these variables. To ascertain the validity of the risk score in predicting survival and treatment response within the immunotherapy cohort, we conducted separate validation analyses across two distinct immunotherapy cohorts. KM analysis demonstrated that patients with a low-risk score exhibited superior OS outcomes. Furthermore, a higher proportion of patients demonstrated responsiveness to treatment within the low-risk score group (Figure 7C, 7D). Additionally, our investigation extended to include datasets pertaining to various ICIs and CAR-T therapy, namely GSE78220, GSE91061, GSE100797, GSE16252, GSE35640, GSE135222, GSE126044, Nathanson, and the VanAllen datasets. Noteworthy is the consistent discrimination observed between high- and low-risk patients across five immunotherapy datasets with survival follow-up, employing the risk score based on ICD. Moreover, the majority of predicted AUC values for treatment responsiveness surpassed 0.6 (Supplementary Figure 3). Waterfall plots (Figure 7E) illustrated mutant occurrences within distinct risk score subgroups. Within LUAD, TP53 emerged as the most frequently mutated gene, predominantly exhibiting missense mutations. Notably, individuals with elevated risk scores manifested heightened mutation probabilities in pivotal genes, notably TP53. Additionally, IC50 values for select chemotherapeutic agents (doxorubicin, cisplatin, docetaxel, etoposide, and gemcitabine) were estimated among LUAD patients. Significantly, individuals categorized as high-risk demonstrated increased chemosensitivity compared to their low-risk counterparts (*p* < 0.05), implying a potentially enhanced response to chemotherapy interventions (Figure 7F).

### Validation of expression of genes involved in signature

**Figure 8:**
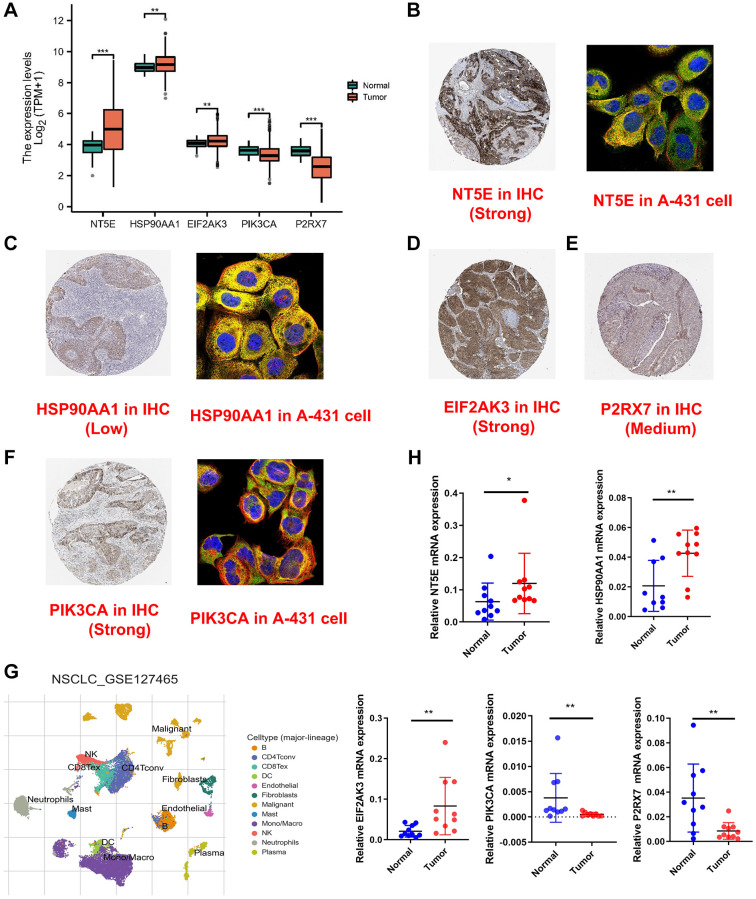
**Validation of expression of genes involved in signature.** (**A**) The expression of the five-signature genes in TCGA database. (**B**–**F**) The cell sublocalization immunofluorescence plot and IHC plot of five-signature genes. (**G**) Graph showing cell clusters identified by using single-cell RNA-seq dataset GSE127465. (**H**) The expression levels of five ICDGs in 10 paired LUAD and matched adjacent normal tissues were examined by qRT-PCR. ^*^p < 0.05, ^**^p < 0.01, ^***^p < 0.001.

The five-gene signature’s expression was validated utilizing the TCGA database, with NT5E, HSP90AA1, and EIF2AK3 demonstrating elevated expression levels in tumor tissues, while PIK3CA and P2RX7 exhibited heightened expression in normal tissues (Figure 8A). Subsequent analysis involved immunofluorescence and IHC imaging to delineate the expression patterns and staining intensities of these genes; however, EIF2AK3 and P2RX7 were not subjected to immunofluorescence analysis (Figure 8B–8F). Moreover, examination of the single-cell dataset revealed significant underexpression of all genes except HSP90AA1 in malignant cells (Figure 8G, Supplementary Figure 4). Subsequent examination of cell communication patterns revealed that malignancies characterized by elevated risk scores exhibited heightened interactions with neighboring cells. Subsequently, we proceeded to assess the clinical relevance of this signature utilizing tissue specimens obtained from the Second Hospital of Hebei Medical University. qRT-PCR was employed to evaluate the messenger RNA (mRNA) expression levels of the signature-associated ICDGs in ten fresh pairs of tissues from patients diagnosed with LUAD and their corresponding adjacent normal lung tissues. As illustrated in Figure 8H, our qRT-PCR analysis of clinical specimens obtained from our institution demonstrated expression profiles consistent with those observed in TCGA database.

## DISCUSSION

The main challenge in current tumor immunotherapy is the low immunogenicity of tumors, the existence of a tumor suppressive immune microenvironment, and a series of immune escape mechanisms by which tumors prevent the body’s immune system from playing its normal anti-tumor role [21, 22]. As an immune-responsive cell death mechanism, ICD is expected to break the immunosuppressive tumor microenvironment, initiate a T-cell-mediated adaptive immune response, and mobilize the systemic immune response for long-term tumor suppression [23–25]. Therefore, it could be advantageous to identify ICD-related biomarkers that help distinguish patients premised on the benefits they would derive from immunotherapy.

In this study, we have demonstrated a robust association between the expression of ICDGs and both the prognosis and the tumor microenvironment of LUAD. Utilizing consensus clustering based on ICDGs expression, we have discerned three distinct ICD subtypes. Notably, subtype B exhibits a favorable clinical prognosis alongside heightened levels of immune cell infiltration. Moreover, tumors classified under subtype B manifest elevated immune, stromal, and ESTIMATE scores compared to other subtypes. This subtype also displays augmented expression of ICI and HLA, as well as a richer array of enrichment pathways compared to subtypes A and C. To further assess the prognostic utility of ICDGs, we have devised an ICD prognostic signature employing a combination of Cox regression and LASSO regression analyses. Among the five identified ICDGs, NT5E, HSP90AA1, and EIF2AK3 exhibited heightened expression levels in tumor tissues, while PIK3CA and P2RX7 demonstrated elevated expression levels in normal tissues. To assess the prognostic efficacy of the developed signature, we stratified patients with LUAD into low- and high-risk categories based on the median risk score. Subsequently, KM survival analysis was conducted, revealing that patients in the high-risk subgroup experienced significantly poorer OS compared to those in the low-risk subgroup across both TCGA-LUAD and GSE68465 cohorts. These findings are in concordance with the outcomes derived from ROC curves. Furthermore, we devised a nomogram by incorporating the risk score and disease stage, aiming to facilitate the clinical application of our findings. The nomogram derived from the risk score exhibited strong predictive capability in both the TCGA-LUAD and GSE68465 cohorts, as evidenced by the calibration curves closely aligning with the true curves. To assess the potential of the risk score as a standalone prognostic marker in LUAD, univariate and multivariate Cox regression analyses were conducted across the TCGA-LUAD and GSE68465 cohorts. Findings indicated that the risk score independently correlates with increased risk in LUAD and surpasses conventional prognostic factors such as age, tumor grade, and stage in prognosticating patient survival outcomes.

Compared to conventional models that may primarily focus on a single aspect of tumor biology, such as genetic mutations or expression profiles (Autophagy related genes, glycolysis related genes, ferroptosis related genes, etc), our model encompasses a broader spectrum by considering the immune landscape and the implications of ICDGs. This approach not only contributes to our understanding of the molecular underpinnings of LUAD but also offers practical insights into patient stratification and potential therapeutic targets. Furthermore, the prognostic signature developed from five key ICDGs in our study provides a novel tool for predicting patient outcomes, surpassing traditional prognostic indicators in accuracy. This signature’s effectiveness is validated across multiple cohorts, underscoring its robustness and potential for clinical application.

In investigating the discriminative potential of risk subgroups for TME and its relevance to immunotherapy, we applied six distinct algorithms to assess immune cell infiltration levels across various samples. As anticipated, the abundance of cytotoxic immune cells, including CD4+ and CD8+ T cells, exhibited a positive correlation with escalating risk scores. Moreover, notable distinctions in immune cell distribution were observed among different risk subgroups. Notably, patients afflicted with subtype B disease displayed a lower risk score. Previous work by Thorsson et al. [15] delineated six immune expression signature subtypes predicated on comprehensive gene expression profiles across all solid tumors in TCGA. Our analysis revealed that the risk score expression was minimal in the C6 subtype and maximal in the C1/C2 subtypes, while the low-risk subgroup exhibited a greater prevalence of C3. Recognizing the pivotal influence of the stemness index on immunotherapeutic outcomes, correlation analysis unveiled a positive association between the risk score and DNAss as well as RNAss. Subsequently, we investigated the relationship between the risk score and the TME score. Our findings indicate a negative association between the risk score and stromal and immune scores, while demonstrating a positive correlation with tumor purity. These results suggest that individuals with low-risk scores exhibit a favorable immune-activated TIME, potentially enhancing the efficacy of immunotherapy. Notably, in a thorough validation analysis across two distinct immunotherapy datasets (IMvigor and PRJEB23709), we observed improved OS and enhanced responses to immunotherapy among patients with low-risk scores. Thus, our study underscores the significance of the risk score as a promising biomarker for assessing immunotherapy efficacy in patients with LUAD by virtue of its strong correlation with the tumor microenvironment.

## CONCLUSIONS

In summary, this study delineated three distinct molecular subtypes in LUAD using ICDGs and formulated a prognostic risk signature based on five ICDGs. Notably, Subtype B and the low-risk subgroups exhibited markedly improved survival outcomes alongside a heightened immune-activated TIME. Moreover, the risk score emerged as a promising biomarker indicative of treatment efficacy in LUAD patients undergoing immunotherapy. These findings contribute valuable insights into the anti-tumor mechanisms of ICD and propose a novel and efficacious immunotherapeutic approach for further exploration in tumor immunology research.

## Supplementary Materials

Supplementary Figures

Supplementary Tables 1 and 2

Supplementary Tables 3 and 4
