# Supplementary material for: Identifying an immunogenic cell death-related gene signature contributes to predicting prognosis, immunotherapy efficacy, and tumor microenvironment of lung adenocarcinoma
Source: Aging (Albany NY). 2024 Apr 3;16(7):6290–313. doi: 10.18632/aging.205705 (PMC11042933; doi:10.18632/aging.205705)
Supplement: Supplementary Figures [file aging-16-205705-s001.pdf]

SUPPLEMENTARY FIGURES

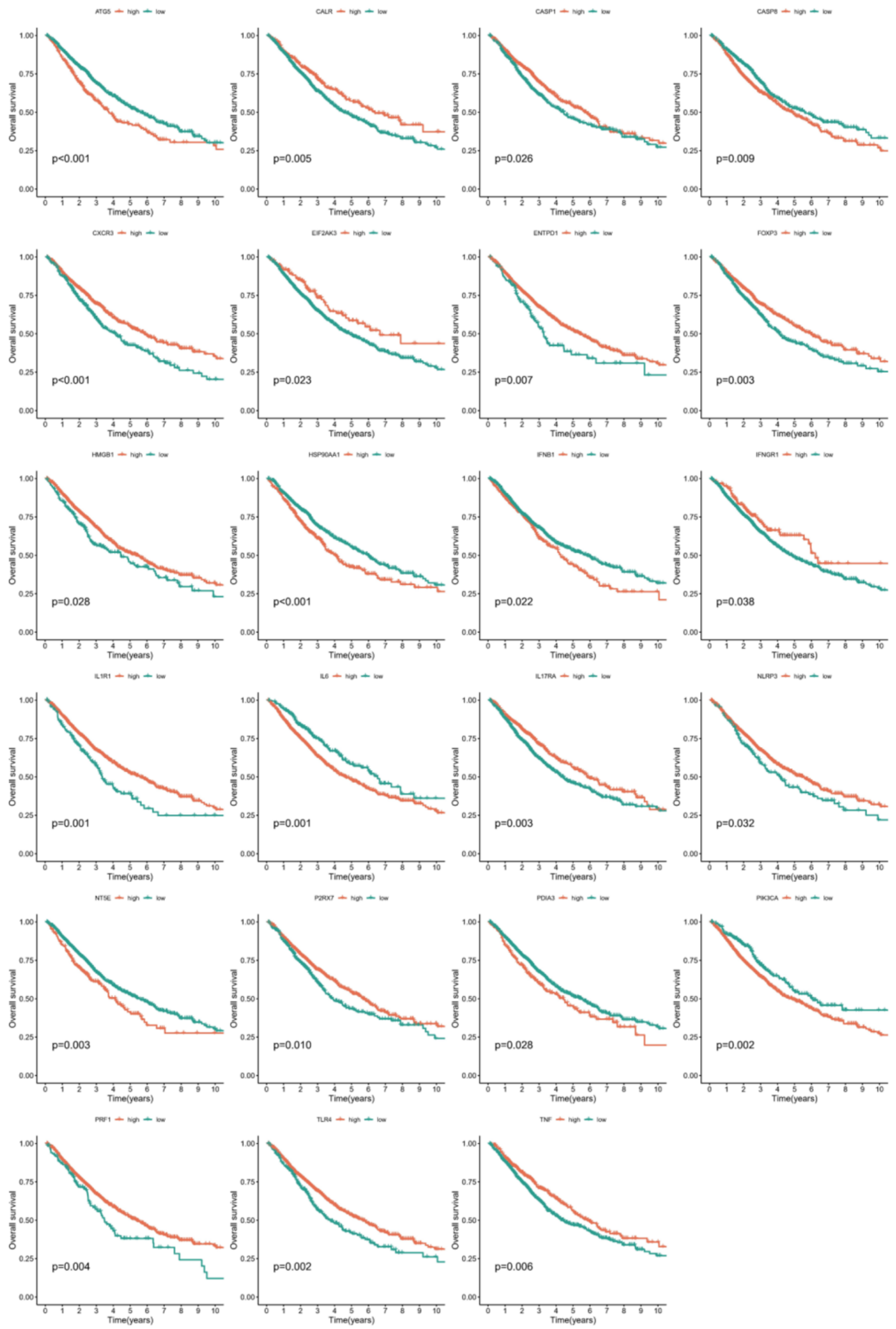

Supplementary Figure 1. The KM analysis of the 23 ICDGs.

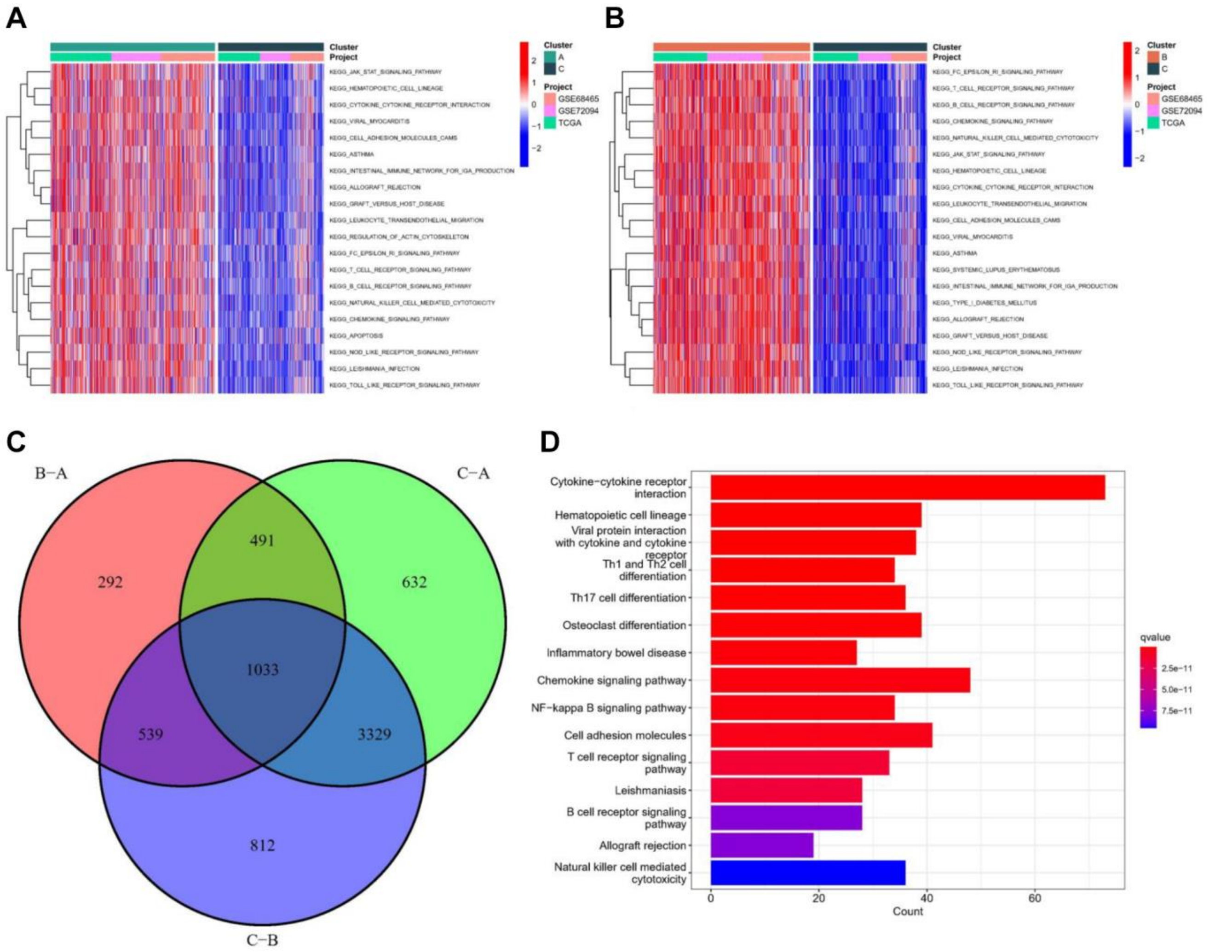

Supplementary Figure 2. Identification and enrichment analysis of differential genes among the subtypes. (**A**, **B**) GSVA of biological pathways between two distinct subtypes. (**C**) Wayne diagram was adopted to screen the difference genes in the three subtypes, and 1033 genes were obtained after taking the intersection. (**D**) KEGG enrichment analysis for DEGs between different molecular subtypes.

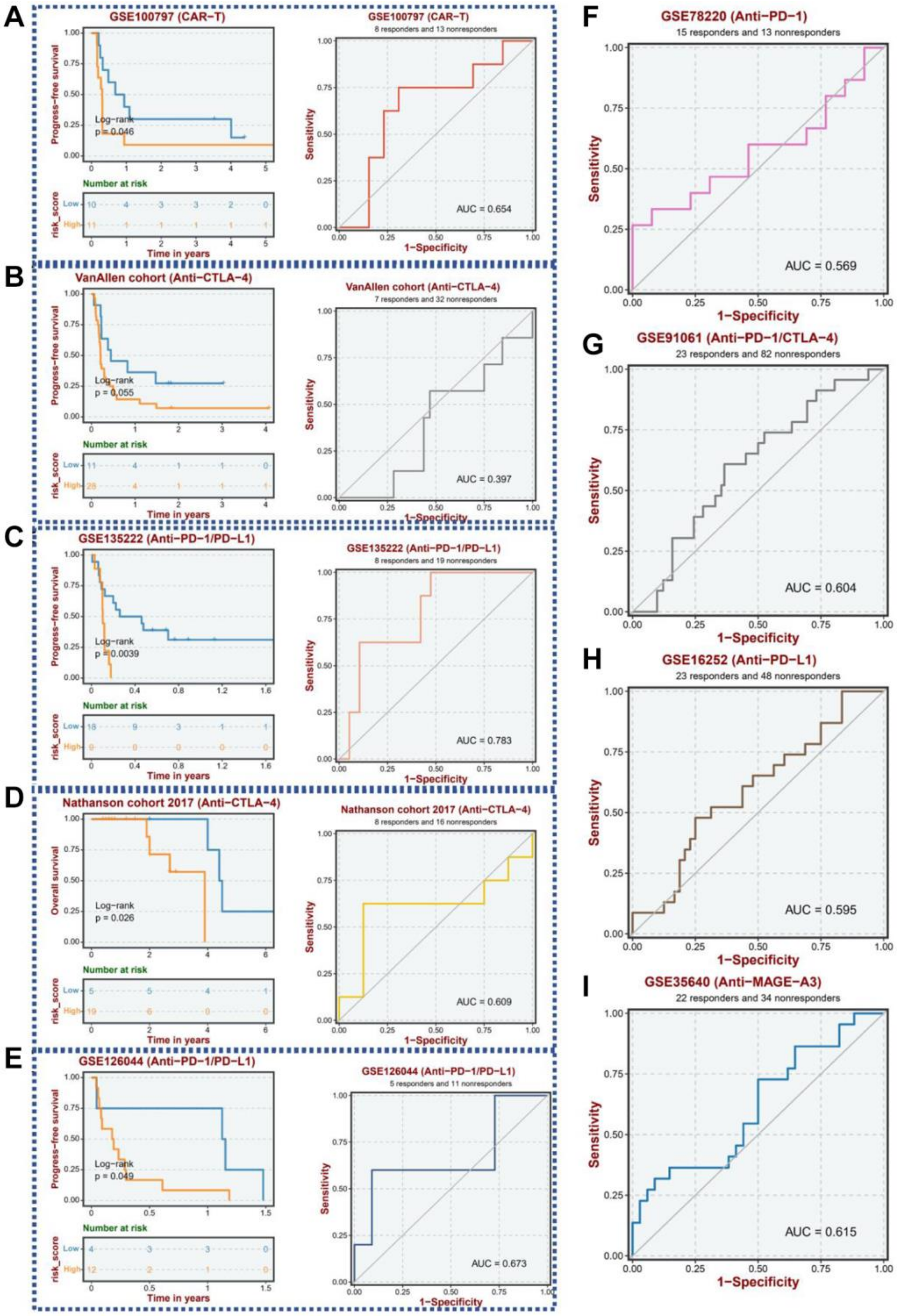

Supplementary Figure 3. The ICD predicts immunotherapeutic benefits. (**A**–**E**) KM curves for patients with high and low ICD in the GSE100797, VanAllen, GSE135222, Nathanson, and GSE126044 cohorts. ROC curve of the ICD in the GSE100797, VanAllen, GSE135222, Nathanson, and GSE126044 cohorts. (**F**–**I**) ROC curve of the ICD in the GSE78220, GSE91061, GSE16252, and GSE35640 cohorts.

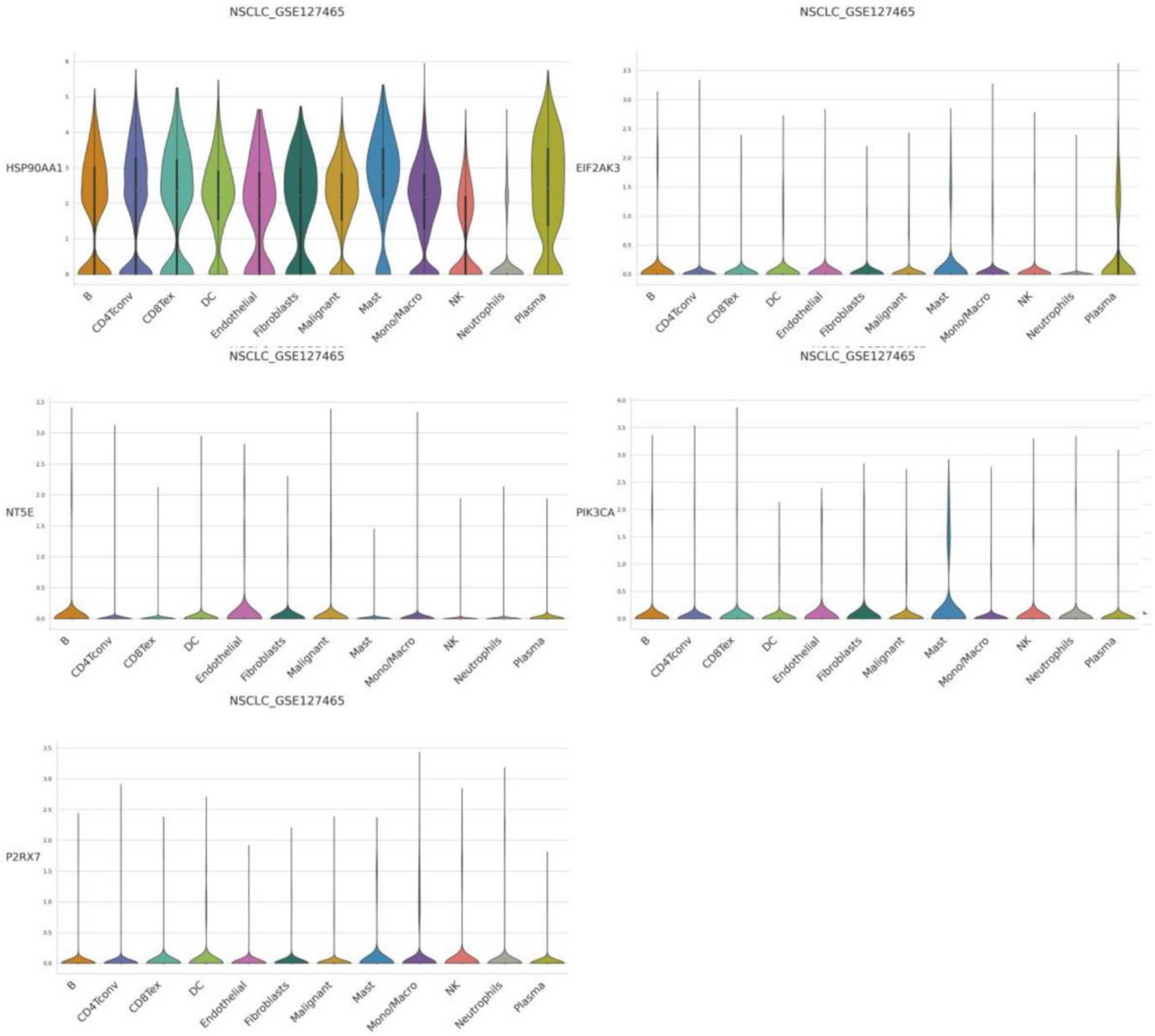

Supplementary Figure 4. Violin plots showing the expression levels of five signature-related ICDGs in the cell clusters identified by the single-cell RNA-seq dataset GSE127465.
