# Supplementary material for: Identifying an immunogenic cell death-related gene signature contributes to predicting prognosis, immunotherapy efficacy, and tumor microenvironment of lung adenocarcinoma
Source: Aging (Albany NY). 2024 Apr 3;16(7):6290–313. doi: 10.18632/aging.205705 (PMC11042933; doi:10.18632/aging.205705)
Supplement: Supplementary Tables 3 and 4 [file aging-16-205705-s003.pdf]

## SUPPLEMENTARY TABLES

**Supplementary Table 3. The activation states of biological pathways in distinct ICD subtypes.**

| ID | Description | GeneRatio | BgRatio | *p*-value | *p*.adjust | *q*-value | geneID | Count |
|---|---|---|---|---|---|---|---|---|
| hsa04060 | Cytokine-cytokine receptor interaction | 73/625 | 295/8142 | 2.82E-20 | 8.89E-18 | 6.98E-18 | | 73 |
| hsa04640 | Hematopoietic cell lineage | 39/625 | 99/8142 | 7.34E-19 | 1.16E-16 | 9.07E-17 | | 39 |
| hsa04061 | Viral protein interaction with cytokine and cytokine receptor | 38/625 | 100/8142 | 8.98E-18 | 9.43E-16 | 7.41E-16 | | 38 |
| hsa04658 | Th1 and Th2 cell differentiation | 34/625 | 92/8142 | 1.35E-15 | 1.06E-13 | 8.32E-14 | | 34 |
| hsa04659 | Th17 cell differentiation | 36/625 | 108/8142 | 8.31E-15 | 5.24E-13 | 4.11E-13 | | 36 |
| hsa04380 | Osteoclast differentiation | 39/625 | 128/8142 | 1.81E-14 | 9.52E-13 | 7.48E-13 | | 39 |
| hsa05321 | Inflammatory bowel disease | 27/625 | 65/8142 | 4.12E-14 | 1.85E-12 | 1.45E-12 | | 27 |
| hsa04062 | Chemokine signaling pathway | 48/625 | 192/8142 | 7.93E-14 | 3.12E-12 | 2.45E-12 | | 48 |
| hsa04064 | NF-kappa B signaling pathway | 34/625 | 104/8142 | 9.03E-14 | 3.16E-12 | 2.48E-12 | | 34 |
| hsa04514 | Cell adhesion molecules | 41/625 | 149/8142 | 1.78E-13 | 5.61E-12 | 4.40E-12 | | 41 |
| hsa04660 | T cell receptor signaling pathway | 33/625 | 104/8142 | 5.49E-13 | 1.57E-11 | 1.23E-11 | | 33 |
| hsa05140 | Leishmaniasis | 28/625 | 77/8142 | 7.29E-13 | 1.91E-11 | 1.50E-11 | | 28 |
| hsa04662 | B cell receptor signaling pathway | 28/625 | 82/8142 | 4.35E-12 | 1.00E-10 | 7.88E-11 | | 28 |
| hsa05330 | Allograft rejection | 19/625 | 38/8142 | 4.46E-12 | 1.00E-10 | 7.88E-11 | | 19 |
| hsa04650 | Natural killer cell mediated cytotoxicity | 36/625 | 131/8142 | 5.79E-12 | 1.22E-10 | 9.55E-11 | | 36 |
| hsa05152 | Tuberculosis | 42/625 | 180/8142 | 3.42E-11 | 6.74E-10 | 5.29E-10 | | 42 |
| hsa05340 | Primary immunodeficiency | 18/625 | 38/8142 | 5.28E-11 | 9.78E-10 | 7.68E-10 | | 18 |
| hsa04672 | Intestinal immune network for IgA production | 20/625 | 49/8142 | 1.27E-10 | 2.23E-09 | 1.75E-09 | | 20 |
| hsa04940 | Type I diabetes mellitus | 18/625 | 43/8142 | 6.63E-10 | 1.10E-08 | 8.63E-09 | | 18 |
| hsa05150 | Staphylococcus aureus infection | 27/625 | 96/8142 | 1.54E-09 | 2.42E-08 | 1.90E-08 | | 27 |
| hsa05323 | Rheumatoid arthritis | 26/625 | 93/8142 | 3.57E-09 | 5.33E-08 | 4.19E-08 | | 26 |
| hsa05332 | Graft-versus-host disease | 17/625 | 42/8142 | 3.72E-09 | 5.33E-08 | 4.19E-08 | | 17 |
| hsa05169 | Epstein-Barr virus infection | 41/625 | 202/8142 | 5.28E-09 | 7.23E-08 | 5.67E-08 | | 41 |
| hsa05166 | Human T-cell leukemia virus 1 infection | 43/625 | 222/8142 | 9.77E-09 | 1.28E-07 | 1.01E-07 | | 43 |
| hsa04630 | JAK-STAT signaling pathway | 34/625 | 162/8142 | 4.65E-08 | 5.86E-07 | 4.60E-07 | | 34 |
| hsa04612 | Antigen processing and presentation | 22/625 | 78/8142 | 4.84E-08 | 5.87E-07 | 4.61E-07 | | 22 |
| hsa04610 | Complement and coagulation cascades | 23/625 | 85/8142 | 5.63E-08 | 6.57E-07 | 5.16E-07 | | 23 |
| hsa05144 | Malaria | 17/625 | 50/8142 | 8.03E-08 | 9.03E-07 | 7.09E-07 | | 17 |
| hsa04145 | Phagosome | 32/625 | 152/8142 | 1.08E-07 | 1.17E-06 | 9.18E-07 | | 32 |
| hsa05320 | Autoimmune thyroid disease | 17/625 | 53/8142 | 2.12E-07 | 2.22E-06 | 1.75E-06 | | 17 |
| hsa05145 | Toxoplasmosis | 26/625 | 112/8142 | 2.26E-07 | 2.30E-06 | 1.81E-06 | | 26 |
| hsa05235 | PD-L1 expression and PD-1 checkpoint pathway in cancer | 22/625 | 89/8142 | 6.14E-07 | 6.04E-06 | 4.74E-06 | | 22 |
| hsa05202 | Transcriptional misregulation in cancer | 35/625 | 193/8142 | 1.23E-06 | 1.18E-05 | 9.24E-06 | | 35 |
| hsa05310 | Asthma | 12/625 | 31/8142 | 1.37E-06 | 1.27E-05 | 9.94E-06 | | 12 |

| | | | | | | | |
|---|---|---|---|---|---|---|---|
| hsa05416 | Viral myocarditis | 17/625 | 60/8142 | 1.52E-06 | 1.37E-05 | 1.08E-05 | 17 |
| hsa05135 | Yersinia infection | 27/625 | 137/8142 | 4.09E-06 | 3.58E-05 | 2.81E-05 | 27 |
| hsa05170 | Human immunodeficiency virus 1 infection | 36/625 | 212/8142 | 4.29E-06 | 3.66E-05 | 2.87E-05 | 36 |
| hsa04010 | MAPK signaling pathway | 45/625 | 294/8142 | 5.09E-06 | 4.22E-05 | 3.31E-05 | 45 |
| hsa05142 | Chagas disease | 22/625 | 102/8142 | 7.02E-06 | 5.67E-05 | 4.45E-05 | 22 |
| hsa04625 | C-type lectin receptor signaling pathway | 22/625 | 104/8142 | 9.78E-06 | 7.70E-05 | 6.05E-05 | 22 |
| hsa04670 | Leukocyte transendothelial migration | 23/625 | 114/8142 | 1.41E-05 | 0.000108 | 8.50E-05 | 23 |
| hsa04620 | Toll-like receptor signaling pathway | 21/625 | 104/8142 | 3.27E-05 | 0.000245 | 0.000192 | 21 |
| hsa04668 | TNF signaling pathway | 22/625 | 112/8142 | 3.34E-05 | 0.000245 | 0.000192 | 22 |
| hsa04664 | Fc epsilon RI signaling pathway | 16/625 | 68/8142 | 4.06E-05 | 0.00029 | 0.000228 | 16 |
| hsa05133 | Pertussis | 17/625 | 76/8142 | 4.71E-05 | 0.000325 | 0.000255 | 17 |
| hsa05162 | Measles | 25/625 | 139/8142 | 4.74E-05 | 0.000325 | 0.000255 | 25 |
| hsa05163 | Human cytomegalovirus infection | 34/625 | 225/8142 | 9.53E-05 | 0.000639 | 0.000502 | 34 |
| hsa04666 | Fc gamma R-mediated phagocytosis | 19/625 | 97/8142 | 0.000118 | 0.000775 | 0.000609 | 19 |
| hsa04014 | Ras signaling pathway | 34/625 | 232/8142 | 0.000175 | 0.001127 | 0.000885 | 34 |
| hsa05417 | Lipid and atherosclerosis | 32/625 | 215/8142 | 0.000203 | 0.001277 | 0.001003 | 32 |
| hsa05164 | Influenza A | 27/625 | 171/8142 | 0.000238 | 0.001473 | 0.001156 | 27 |
| hsa05221 | Acute myeloid leukemia | 14/625 | 67/8142 | 0.000459 | 0.002783 | 0.002185 | 14 |
| hsa04613 | Neutrophil extracellular trap formation | 27/625 | 190/8142 | 0.001281 | 0.007613 | 0.005979 | 27 |
| hsa04210 | Apoptosis | 21/625 | 136/8142 | 0.001524 | 0.00867 | 0.006809 | 21 |
| hsa05322 | Systemic lupus erythematosus | 21/625 | 136/8142 | 0.001524 | 0.00867 | 0.006809 | 21 |
| hsa05143 | African trypanosomiasis | 9/625 | 37/8142 | 0.001541 | 0.00867 | 0.006809 | 9 |
| hsa05171 | Coronavirus disease - COVID-19 | 31/625 | 232/8142 | 0.00162 | 0.008953 | 0.007031 | 31 |
| hsa04657 | IL-17 signaling pathway | 16/625 | 94/8142 | 0.001949 | 0.010583 | 0.008311 | 16 |
| hsa04611 | Platelet activation | 19/625 | 124/8142 | 0.002742 | 0.014639 | 0.011496 | 19 |
| hsa04621 | NOD-like receptor signaling pathway | 25/625 | 184/8142 | 0.003536 | 0.018563 | 0.014578 | 25 |
| hsa05161 | Hepatitis B | 22/625 | 162/8142 | 0.006041 | 0.031193 | 0.024496 | 22 |
| hsa04151 | PI3K-Akt signaling pathway | 40/625 | 354/8142 | 0.008194 | 0.041631 | 0.032693 | 40 |
| hsa05134 | Legionellosis | 10/625 | 57/8142 | 0.010598 | 0.052991 | 0.041614 | 10 |
| hsa04142 | Lysosome | 18/625 | 132/8142 | 0.011819 | 0.05805 | 0.045586 | 18 |
| hsa04936 | Alcoholic liver disease | 19/625 | 142/8142 | 0.011979 | 0.05805 | 0.045586 | 19 |
| hsa05167 | Kaposi sarcoma-associated herpesvirus infection | 24/625 | 194/8142 | 0.013246 | 0.06322 | 0.049647 | 24 |

**Supplementary Table 4. Prognostic analysis of 5 subtype-associated genes with a multifactorial Cox regression analysis.**

| ID | Coef | HR | HR.95L | HR.95H | *P*-value |
|---|---|---|---|---|---|
| NT5E | 0.1087 | 1.1148 | 1.003 | 1.239 | 0.0438 |
| HSP90AA1 | 0.4088 | 1.505 | 1.0901 | 2.0779 | 0.013 |
| EIF2AK3 | −0.5949 | 0.5516 | 0.4097 | 0.7426 | 0.0001 |
| PIK3CA | 0.5066 | 1.6597 | 1.1893 | 2.3161 | 0.0029 |
| P2RX7 | −0.3506 | 0.7043 | 0.5827 | 0.8512 | 0.0003 |
